# Supplementary material for: Polyurethane-Encapsulated Biomass Films Based on MXene@Loofah Sponge for Piezoresistive Pressure Sensor Applications
Source: Polymers (Basel). 2024 May 12;16(10):1377. doi: 10.3390/polym16101377 (PMC11125038; doi:10.3390/polym16101377)
Supplement: Supplementary file 1 [file polymers-16-01377-s001.zip › polymers-2966352-supplementary.pdf]

Support Information

Polyurethane-Encapsulated Biomass Films Based on MXene@Loofah Sponges for Piezoresistive Pressure Sensor Applications

Qihan Jia, Shuai Liu and Haibo Wang *

College of Biomass Science and Engineering, Sichuan University, Chengdu 610065, China; jiaqihan@stu.scu.edu.cn (Q.J.); 2021323080023@stu.scu.edu.cn (S.L.)
* Correspondence: whb6985@scu.edu.cn; Tel.: +86-28-85401296

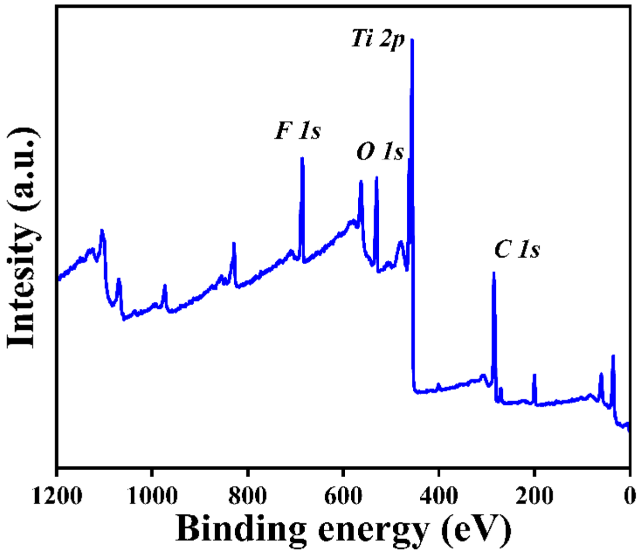

Figure S1. X-ray photoelectron spectroscopy (XPS) spectrum of MXene

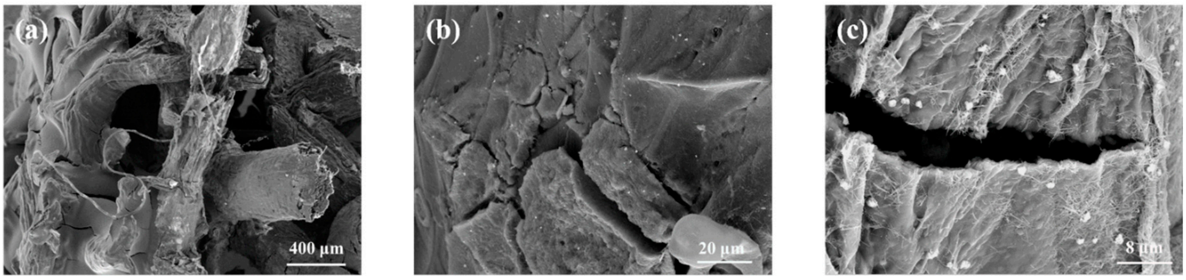

Figure S2. SEM images of PAM@Loofah (a) cross-section and (b,c) corresponding enlarged image.

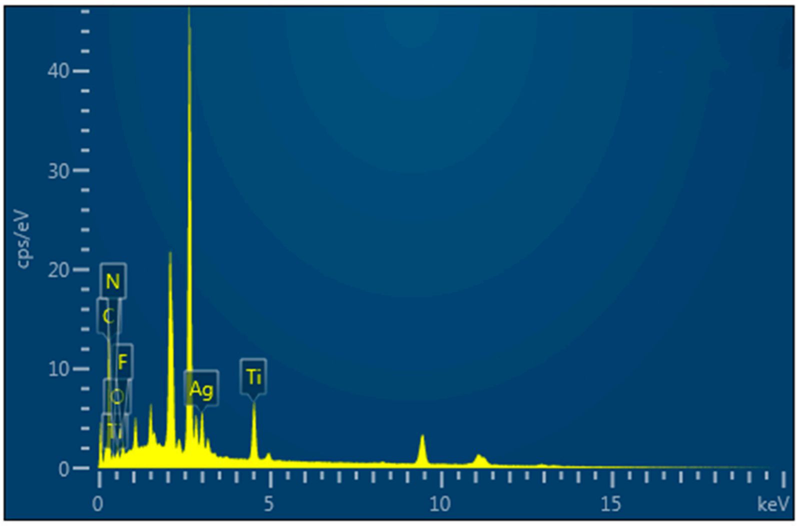

Figure S3. EDS image of the content of each element in PAM@loofah

Table S1. Content of each element in PAM@Loofah

Elements	Wt%	Atomic Percentage (%)
C	38.37	57.67
N	8.36	10.77
O	12.9	14.56
F	8.81	8.37
Ti	16	6.03
Ag	15.56	2.6
